# Pathophysiological aspects of hyperglycemia in children with meningococcal sepsis and septic shock: a prospective, observational cohort study

**DOI:** 10.1186/cc10006

**Published:** 2011-01-31

**Authors:** Jennifer J Verhoeven, Marieke den Brinker, Anita CS Hokken-Koelega, Jan A Hazelzet, Koen FM Joosten

**Affiliations:** 1 Department of Intensive Care, Erasmus MC - Sophia Children's Hospital, Dr. Molewaterplein 60, Rotterdam, 3015 GJ, The Netherlands; 2 Department of Pediatrics, Ghent University Hospital, De Pintelaan 185, Ghent, 9000, Belgium; 3 Department of Pediatric Endocrinology, Erasmus MC - Sophia Children's Hospital, Dr. Molewaterplein 60, Rotterdam, 3015 GJ, The Netherlands

## Abstract

**Introduction:**

The objective of this study was to investigate the occurrence of hyperglycemia and insulin response in critically ill children with meningococcal disease in the intensive care unit of an academic children's hospital.

**Methods:**

Seventy-eight children with meningococcal disease were included. The group was classified into shock non-survivors, shock survivors and sepsis survivors. There were no sepsis-only non-survivors. The course of laboratory parameters during 48 hours was assessed. Insulin sensitivity and β-cell function on admission were investigated by relating blood glucose level to insulin level and C-peptide level and by homeostasis model assessment (HOMA) [β-cell function (HOMA-%B) and insulin sensitivity (HOMA-%S)].

**Results:**

On admission, hyperglycemia (glucose >8.3 mmol/l) was present in 33% of the children. Shock and sepsis survivors had higher blood glucose levels compared with shock non-survivors. Blood glucose level on admission correlated positively with plasma insulin, C-peptide, cortisol, age and glucose intake. Multiple regression analysis revealed that both age and plasma insulin on admission were significantly related to blood glucose. On admission, 62% of the hyperglycemic children had overt insulin resistance (glucose >8.3 mmol/l and HOMA-%S <50%); 17% had β-cell dysfunction (glucose >8.3 mmol/l and HOMA-%B <50%) and 21% had both insulin resistance and β-cell dysfunction. Hyperglycemia was present in 11% and 8% of the children at 24 and 48 hours after admission, respectively.

**Conclusions:**

Children with meningococcal disease often show hyperglycemia on admission. Both insulin resistance and β-cell dysfunction play a role in the occurrence of hyperglycemia. Normalization of blood glucose levels occurs within 48 hours, typically with normal glucose intake and without insulin treatment.

## Introduction

Critical illness is associated with many endocrine and metabolic changes, including changes in the glucose homeostasis [1-7]. Both hypoglycemia and hyperglycemia may lead to adverse outcome as expressed in length of pediatric intensive care unit (PICU) stay and mortality rates [6-16].

A follow-up study in patients who survived meningococcal septic shock in childhood showed that severe mental retardation was associated with hypoglycemia during admission [17]. Children who died from meningococcal septic shock appeared to have significantly lower levels of blood glucose on admission to the PICU in comparison with those who survived, in whom levels were moderately increased [4,5]. The most severely ill children had signs of (relative) adrenal insufficiency on admission. Deficiency of substrate, reduced activity of adrenal enzymes because of endotoxins, cytokines, or medication, and shock with disseminated intravascular thrombosis can cause necrosis of the adrenal glands and result in (relative) adrenal insufficiency in children with meningococcal disease [5].

Many children with meningococcal septic shock suffer from hyperglycemia [12,18,19]. The pathophysiological mechanism leading to hyperglycemia in critically ill children with meningococcal disease may be different from that in adults. Recently, it was shown that the acute phase of sepsis in children is quite different from that in adults [18]. It was suggested that hyperglycemia associated with β-cell dysfunction rather than insulin resistance may be the normal pathophysiological response in children with meningococcal septic shock. It was also suggested that treatment of hyperglycemia with exogenous insulin may not be supportive and may even be potentially detrimental in critically ill children [18].

Better insight into pathophysiological mechanisms leading to hyperglycemia is crucial to improve treatment strategies. The gold standard for quantifying insulin sensitivity *in vivo *is the hyperinsulinemic euglycemic clamp technique [20]. This is a complex and invasive technique and therefore is not easily applied in studies with critically ill children. The search for uncomplicated and inexpensive quantitative tools to evaluate insulin sensitivity has led to the development of other assessments. The fasting glucose-to-insulin ratio and homeostasis model assessment (HOMA) of insulin resistance have been proven to be useful estimates of insulin sensitivity, also in critical illness [21-24]. There is a good correlation between estimates of insulin resistance derived from HOMA and from the hyperinsulinemic euglycemic clamp [24]. The assessment of β-cell function is difficult because the β-cell response to the secretory stimuli is complex. There is no gold standard for β-cell function. The HOMA method for assessing β-cell function (HOMA-%B) is based on measurements of fasting insulin or C-peptide concentration to calculate pre-hepatic insulin secretion in relation to blood glucose levels [24]. The objective of the present study was to investigate the occurrence of hyperglycemia in relation to the insulin response and exogenous factors, such as glucose intake and drug use, in a homogenous group of critically ill children with meningococcal sepsis or meningococcal septic shock or both.

## Materials and methods

### Patients

The study population consisted of previously healthy children who were admitted to the PICU of the Erasmus MC-Sophia Children's Hospital between October 1997 and May 2004 and who were suffering from meningococcal sepsis (that is, sepsis with petechiae/purpura). Sepsis was defined as a body temperature of less than 36.0°C or more than 38.5°C with tachycardia and tachypnea [5]. Children were determined to have septic shock if they had persistent hypotension or evidence of poor end-organ perfusion, defined as at least two of the following: (a) unexplained metabolic acidosis (pH of less than 7.3 or base excess of not more than 5 mmol/L or plasma lactate levels of greater than 2.0 mmol/L), (b) arterial hypoxia (partial pressure of oxygen [PO_2_] of less than 75 mm Hg, a PO_2_/fraction of inspired oxygen [FiO_2_] ratio of less than 250 or transcutaneous oxygen saturation of less than 96%) in patients without overt cardiopulmonary disease, (c) acute renal failure (diuresis of less than 0.5 mL/kg per hour for at least 1 hour despite acute volume loading or evidence of adequate intravascular volume without pre-existing renal disease), or (d) sudden deterioration of the baseline mental status [5]. Sepsis or septic shock was diagnosed in the children within the first hours after admission to the PICU.

Children were not eligible for the study if they had pre-existing diabetes mellitus or had received radiation or chemotherapy within the previous 6 months. Thirty-five of the included 78 children participated in a randomized, double-blinded, placebo-controlled study. They received either placebo or activated protein C concentrate (APC) starting after admission, every 6 hours for the first days of admission, and then every 12 hours to a maximum of 7 days [19]. APC is assumed not to influence the endocrine and metabolic assays [5]. The Erasmus MC Medical Ethics Review Board approved the study, and written informed consent was obtained from the parents or legal representatives.

### Clinical parameters

Disease severity was assessed by the pediatric risk of mortality (PRISM II) score on the day of admission. In those who died within 24 hours after PICU admission, a PRISM score of the first 6 hours was calculated [25]. Glucocorticoid administration, inotropic medication, and use of mechanical ventilation were recorded. Equivalent doses of prednisolone, expressed per body weight (milligrams per kilogram), were calculated, using the glucocorticoid equivalents of 20, 5, and 0.75 mg for hydrocortisone, prednisolone, and dexamethasone, respectively. Inotropic support was quantified by the vasopressor score developed by Hatherill and colleagues [26].

### Nutrition

The children were fed enterally or parenterally (or both) according to a standard feeding protocol as previously described [27]. If enteral feeding could not be started on the second day, parenteral feeding was started. On admission at the PICU, glucose was administered at a rate of 2 to 6 mg/kg per minute, depending on weight. The initial dose of proteins was 1.0 g/kg per day and that of lipids was 1.0 g/kg per day. If clinically possible, nutrition was adjusted to the normal needs according to dietary reference intakes for healthy children on days 3 and 4.

### Collection of blood and assays

Arterial blood samples for the determination of blood glucose levels and plasma levels of insulin, C-peptide, cortisol, cytokines, C-reactive protein (CRP), lactate, and free fatty acids (FFAs) were collected on admission and at 24 and 48 hours thereafter. Assays were used in accordance with the instructions of the manufacturer. Arterial glucose and lactate were determined on a blood gas analyzer (ABL 625; Radiometer A/S, Copenhagen, Denmark). Hypoglycemia was defined as a blood glucose level of not more than 2.2 mmol/L, and hyperglycemia was defined as a blood glucose level of greater than 8.3 mmol/L [28]. To convert millimoles per liter of glucose to milligrams per deciliter, multiply by 18. The reference level for lactate was less than 2.0 mmol/L. Serum insulin was measured by a two-site chemiluminescent immunometric assay (Immulite 2000; Diagnostics Product Corporation, now part of Siemens, Los Angeles, CA, USA) with a minimum detection level of 35 pmol/L and a maximum fasting reference value of 180 pmol/L. Serum C-peptide was measured by a chemiluminescent immunometric method (Immulite 2000). For children under the age of 13 years, the reference interval ranged between 0.2 and 2.6 nmol/L (0.6 to 7.8 ng/mL) and for children older than 13 years between 0.4 and 2.6 nmol/L (1.3 to 7.9 ng/mL) [29]. Serum cortisol concentrations were determined with a competitive luminescence immunoassay (Immulite 2000). The detection limits of this assay range from 3 to 1,380 nmol/L. Adrenal insufficiency in case of catecholamine-resistant septic shock is assumed at a random total cortisol level of less than 496 nmol/L (less than 18 μg/dL) [30]. FFA was determined by the enzymatic method (Nefac-kit, Wako; Instruchemie BV, Delfzijl, The Netherlands). CRP was determined by immunoturbidimetric assay (normal of less than 2 mg/L) and examined on a 912 analyzer (Roche Diagnostics GmbH, Mannheim, Germany). Cytokine levels were analyzed with an enzyme-linked immunosorbent assay (Sanquin, Amsterdam, The Netherlands). The detection limit of interleukin-6 (IL-6) (lowest positive standard) was 10 pg/mL. The detection limit of tumor necrosis factor-alpha was 5 pg/mL [31].

### Outcome measurements

The total sample was divided into three groups: shock non-survivors, shock survivors, and sepsis survivors, as we have previously reported striking differences in endocrinological and metabolic responses between survivors and non-survivors [5]. The courses of the main endocrinological, metabolic, and immunological laboratory parameters during the first 48 hours of PICU stay were assessed.

The insulin response to hyperglycemia was assessed by investigating insulin response to glucose and by HOMA modeling [24]. The updated HOMA2 computer model was used to determine insulin sensitivity (%S) and β-cell function (%B) from paired plasma glucose and insulin and C-peptide concentrations on admission. Children were considered to be fasting until admission with subsequently only a continuous glucose infusion without enteral intake for more than 6 hours. Determinations of insulin sensitivity and β-cell function were made on admission only.

### Statistical analysis

Analysis was performed with the SPSS statistical software package for Windows (version 16.0; SPSS, Inc., Chicago, IL, USA). Results are expressed as medians and interquartile ranges, unless specified otherwise. Between-group comparisons were made with the Mann-Whitney *U *test for continuous data. The chi-square test was used for comparison of nominal data. The Spearman's correlation coefficient was used to evaluate the relationship between different parameters. Multiple linear regression analysis was applied to evaluate the relationship between admission hyperglycemia and various variables. Data were log-transformed for multiple linear regression analysis when necessary. *P *values of less than 0.05 are considered statistically significant.

## Results

### Patient characteristics

Seventy-eight children (32 females) admitted to our PICU with meningococcal disease were included (Table 1). Their median age was 3.5 years (1.6 to 9.4 years). Blood cultures revealed *Neisseria meningitidis *in 65 children, and meningococcal disease was diagnosed in 13 children on the basis of their typical clinical picture. Sixty-seven children were classified as having meningococcal septic shock, and 11 were classified as having meningococcal sepsis. Nine children with shock died within 24 hours after PICU admission, and 1 child with shock died within 48 hours.

**Table 1:** Patient characteristics on admission

	Shock non-survivors	Shock survivors	Sepsis survivors
Number	10	57	11
Females/Males	2/8	24/33	6/5
Age, years	1.1 (0.6-2.2)^a,b^	4.1 (1.8-9.3)^c^	6.1 (2.8-11.4)^c^
PRISM score	31 (25-35)^d,e^	21 (16-28)^e,f^	9 (8-11)^d,f^
Inotropic medication, number (percentage)	10 (100%)	57 (100%)	2 (18%)
Vasopressor score	3 (3-3)	2 (1-3)	0 (0-1)
Mechanical ventilation, number (percentage)	10 (100%)	37 (65%)	2 (18%)
Steroid treatment, number (percentage)	2 (20%)	6 (11%)	1 (9%)
Prednisolone equivalents, mg/kg	0.9 (0.2-1.6)	2.4 (0.6-4.5)	1.0
Glucose intake, mg/kg per minute	3.3 (0-5.8)	3.9 (1.4-5.0)	1.1 (0.6-3.1)

Data are expressed as median (25th-75th percentile) unless indicated otherwise. The vasopressor score was developed by Hatherill and colleagues [26]. ^a^compared with shock survivors, *P *< 0.05; ^b^compared with sepsis survivors, *P *< 0.05; ^c^compared with shock non-survivors, *P *< 0.05; ^d^compared with shock survivors, *P *< 0.001; ^e^compared with sepsis survivors, *P *< 0.001; ^f^compared with shock non-survivors, *P *< 0.001. PRISM, pediatric risk of mortality.

The total sample was classified into three groups: shock non-survivors (*n *= 10), shock survivors (*n *= 57), and sepsis survivors (*n *= 11). All children with sepsis survived. Shock non-survivors were significantly younger than shock survivors and sepsis survivors (*P *< 0.01). Shock survivors stayed a median of 4.1 days (2.7 to 8.9 days) in the PICU; sepsis survivors stayed a median of 1.1 days (1.0 to 1.9 days) (*P *< 0.001).

### Clinical parameters

Clinical parameters are depicted in Table 1. Median PRISM score was 20 (14 to 29). PRISM scores and IL-6 levels for shock non-survivors were significantly higher than those for both groups of survivors (*P *< 0.001), and those for shock survivors were significantly higher than those for sepsis survivors (*P *< 0.001). APC administration did not influence cortisol levels or coagulation profile (data not shown). Concomitant therapy included antibiotics and administration of fluids in all children. Forty-nine children were mechanically ventilated, and 69 children received inotropic support. Thirty-five children were intubated with a single dose of etomidate. Indications for steroid use were catecholamine-resistant septic shock, with or without hypoglycemia, and meningitis. Nine children received glucocorticoids (hydrocortisone or dexamethasone) just before admission to the PICU; eight of them had catecholamine-resistant septic shock and one had sepsis with meningitis. During admission, another six children with septic shock received steroids (hydrocortisone) because of catecholamine-resistant septic shock. One child experienced severe hyperglycemia (glucose of greater than 20 mmol/L) after PICU admission, was treated with insulin, and was excluded from further analysis after admission. The other children did not receive insulin treatment.

### Nutrition and glucose intake

On admission, median glucose intake was 2.8 mg/kg per minute (1.0 to 5.0 mg/kg per minute), which was not significantly different between shock non-survivors, shock survivors, and sepsis survivors (Table 1). Twenty-four hours after admission, median glucose intake in shock survivors was 5.2 mg/kg per minute (4.3 to 6.4 mg/kg per minute); 48 hours after admission, it was 4.4 mg/kg per minute (3.7 to 6.3 mg/kg per minute). Most sepsis survivors were on a partial oral diet at 24 hours after admission, and this made it difficult to calculate the exact glucose intake.

### Blood analysis

#### Time course

The time course of laboratory parameters is depicted in Table 2. On admission, 26 of the children (33%) were hyperglycemic: 1 shock non-survivor, 19 shock survivors, and 6 sepsis survivors. One child (a shock survivor) was hypoglycemic. In general, shock survivors and sepsis survivors had significantly higher blood glucose levels on admission compared with shock non-survivors. Hyperglycemia was present in 5 shock survivors and 1 shock non-survivor after 24 hours (11%) and in 3 shock survivors after 48 hours (8%). Cortisol and cytokine levels decreased to normal levels within 24 hours.

**Table 2:** Laboratory parameters on admission and at 24 and 48 hours

	Shock non-survivors	Shock survivors			Sepsis survivors	
			
	**T**_**0**_	**T**_**0**_	**T**_**24**_^**g**^	**T**_**48**_^**g**^	**T**_**0**_	**T**_**24**_
	(*n *= 10)	(*n *= 57)	(*n *= 48)	(*n *= 36)	(*n *= 11)	(*n *= 6)
Glucose, mmol/L	4.9^a,b^	7.2^,b,c^	6.7	5.9	8.8^a,c^	6.6
	(2.7-7.0)	(5.3-9.0)	(5.9-7.8)	(5.3-6.6)	(7.5-10.5)	(4.7-7.1)
Insulin, pmol/L	<35^a,b^	101^c^	111	89	104^c^	136
	(<35-57)	(35-197)	(71-169)	(61-157)	(52-226)	(51-236)
C-peptide, nmol/L	-	1.1	2.0	1.5	1.0	1.7
		(0.6-2.7)	(1.0-3.0)	(1.0-1.9)	(0.5-1.8)	(1.0-2.6)
Cortisol but not glucocorticoids, nmol/L	615^a,b^	954^c^	603	554	1,140^c^	447
	(510-930)	(713-1,241)	(430-1,409)	(501-927)	(1,066-1,409)	(263-657)
FFAs, mmol/L	0.3	0.8	0.6	0.3	0.6	0.5
	(0.2-0.5)	(0.5-1.1)	(0.4-0.8)	(0.3-0.6)	(0.5-0.7)	(0.4-0.7)
Lactate, mmol/L	6.8^d,e^	3.7^e,f^	2.0	1.6	2.1^d,f^	0.8
	(5.1-8.0)	(2.6-5.4)	(1.5-2.8)	(1.2-2.3)	(1.6-2.7)	(0.7-0.9)
CRP, mg/L	34^a,e^	89^c^	229	223	75^f^	236
	(23-41)	(59-131)	(181-274)	(159-301)	(36-191)	(195-273)
IL-6, pg/mL	120 × 10^4d,f^	3.5 × 10^4e,f^	0.02 × 10^4b^	0.01 × 10^4^	0.04 × 10^4d,f^	17^a^
	(70-160 × 10^4^)	(1-16 × 10^4^)	(0.01-0.2 × 10^4^)	(0.003-0.03 × 10^4^)	(82-1 × 10^4^)	(<10-0.02 × 10^4^)
TNF-α, pg/mL	42^d^	6^f^	4	-	-	3
	(20-127)	(<5-10.5)	(1-12)			(1-10)

Children who received steroids before or on admission were excluded from determination of median cortisol levels. Data are expressed as median (25th-75th percentile). ^a^Compared with shock survivors, *P *< 0.05; ^b^compared with sepsis survivors, *P *< 0.05; ^c^compared with shock non-survivors, *P *< 0.05; ^d^compared with shock survivors, *P *< 0.001; ^e^compared with sepsis survivors, *P *< 0.001; ^f^compared with shock non-survivors, *P *< 0.001; ^g^one patient with insulin therapy was excluded. CRP, C-reactive protein; FFA, free fatty acid; IL-6, interleukin-6; T_0_, on admission; T_24_, at 24 hours after admission; T_48_, at 48 hours after admission; TNF-α, tumor necrosis factor-alpha.

#### Insulinemic response

##### Association between glucose and insulin

**Figure 1:**
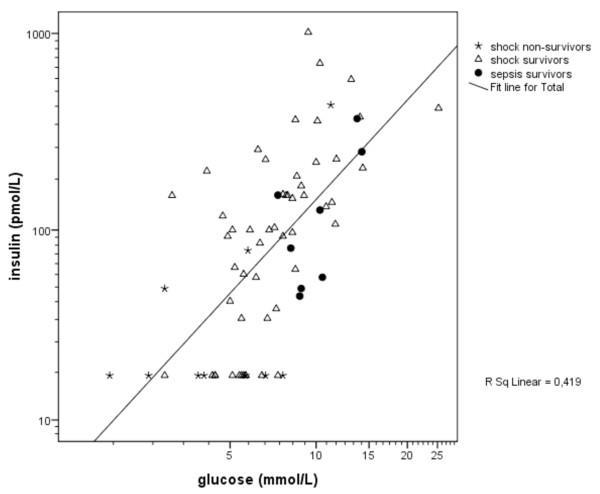
**Relationship between plasma insulin levels and blood glucose levels on admission in shock non-survivors, shock survivors, and sepsis survivors (*r *= 0.67, *P *< 0.001)**.

In Figure 1, the association between glucose and insulin levels is shown for the three groups. Hyperglycemic children had significantly higher insulin levels (214 pmol/L, 128 to 375 pmol/L) and C-peptide levels (1.9 nmol/L, 0.8 to 3.7 nmol/L) in comparison with normoglycemic children (insulin 57 pmol/L, 18 to 101 pmol/L; C-peptide 0.7 nmol/L, 0.3 to 1.6 nmol/L; *P *< 0.001 and *P *= 0.02, respectively).

##### Influence of glucose infusion on insulinemic response

**Figure 2:**
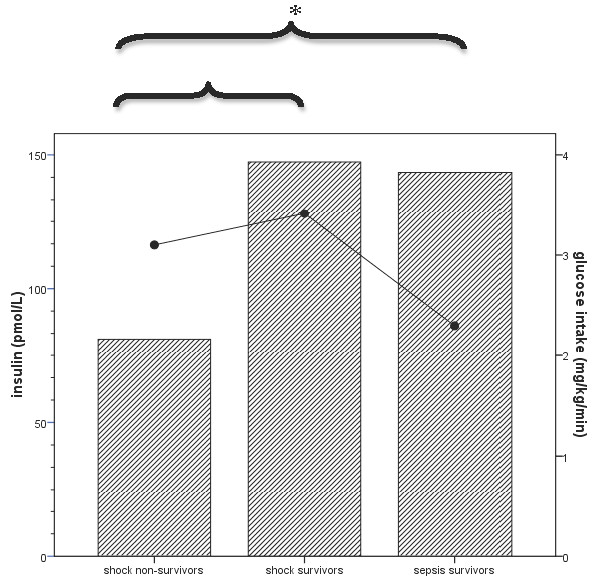
**Mean glucose intake rates and insulin levels on admission in shock non-survivors, shock survivors, and sepsis survivors**. Bars represent mean insulin levels, and dots represent glucose intake rates. Insulin levels in shock survivors and sepsis survivors were significantly higher than in shock non-survivors (*P < 0.05). There were no differences in glucose intake between the patient categories.

Because blood glucose levels and endogenous insulin production are related to exogenous glucose administration, we assessed intravenous glucose infusion rates at the times when blood glucose and insulin levels were drawn (Figure 2). All children received parenteral glucose infusions without enteral intake on admission. Glucose intake rates were not significantly different between children with normoglycemia and those with hyperglycemia (2.4 mg/kg per minute, 0.8 to 5.0 mg/kg per minute versus 4.0 mg/kg per minute, 1.5 to 6.1 mg/kg per minute, respectively; *P *= 0.14) or between shock non-survivors, shock survivors, and sepsis survivors (Table 1).

##### Homeostasis model assessment

**Figure 3:**
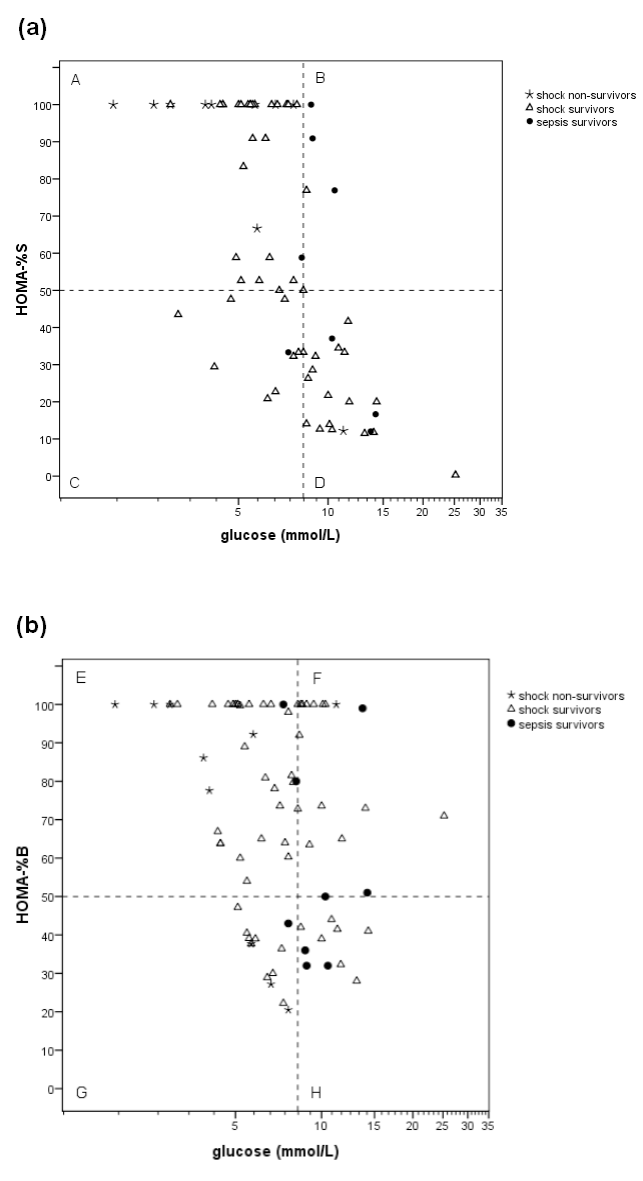
**Homeostasis model assessment and blood glucose levels on admission in shock non-survivors, shock survivors, and sepsis survivors**. **(a) **Homeostatis model assessment of insulin sensitivity (HOMA-%S). The vertical, x-axis reference line represents the limit for normoglycemia (8.3 mmol/L). The horizontal, y-axis reference line represents 50% of maximum insulin sensitivity. **(b) **Homeostatis model assessment of β-cell function (HOMA-%B). The vertical, x-axis reference line represents the limit for normoglycemia (8.3 mmol/L). The horizontal, y-axis reference line represents 50% of maximum β-cell function.

**Figure 4:**
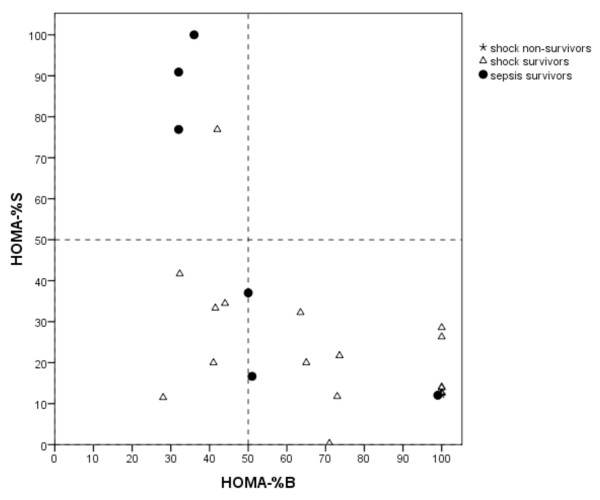
**HOMA-%B plotted against HOMA-%S for hyperglycemic shock non-survivors, shock survivors, and sepsis survivors on admission**. HOMA-%B, homeostatis model assessment of β-cell function; HOMA-%S, homeostatis model assessment of insulin sensitivity.

To determine the occurrence of insulin resistance and decreased β-cell function in hyperglycemic children, HOMA-%S and HOMA-%B were calculated. Paired insulin and glucose levels were used to calculate HOMA-%S. Paired C-peptide (*n *= 35) or insulin (*n *= 43) levels and glucose levels were used to calculate HOMA-%B. In Figure 3, glucose and HOMA are plotted for the three groups. Figure 3a shows the plot of glucose levels and insulin sensitivity (HOMA-%S); Figure 3b shows the plot of glucose levels and β-cell function (HOMA-%B). The scatter plots are divided into four zones by the x-axis reference line representing the maximum reference level for normoglycemia (glucose of 8.3 mmol/L, 150 mg/dL) and a y-axis reference line at 50% of normal insulin sensitivity (Figure 3a) or at 50% of normal β-cell function (Figure 3b). Zone D represents children with hyperglycemia and insulin resistance; zone H represents children with hyperglycemia and β-cell dysfunction. Figure 3a (zone C) shows that insulin resistance also occurred in the children with blood glucose levels of below 8.3 mmol/L but less frequently than in the hyperglycemic children. Sixty-two percent of hyperglycemic children were insulin-resistant, 17% had β-cell dysfunction, and 21% had both insulin resistance and β-cell dysfunction (Figure 4).

#### Influence of exogenous factors on glucose homeostasis

##### Influence of glucocorticoids

Nine children were treated with glucocorticoids just before admission. They tended to have higher blood glucose (8.4 mmol/L, 5.4 to 12.4 mmol/L) and cortisol (1,308 nmol/L, 615 to 2,094 nmol/L) levels on admission in comparison with the other children (glucose 7.2 mmol/L, 5.3 to 8.9 mmol/L and cortisol 955 nmol/L, 666 to 1,201 nmol/L), but these differences were not significant (*P *= 0.18 and *P *= 0.22, respectively). After admission, an additional six children were treated with hydrocortisone (prednisolone equivalent dose of 1.6 mg/kg, 0.5 to 3.1 mg/kg) within 24 hours. At 24 hours after admission, cortisol levels (1,824 nmol/L, 270 to 8,490 nmol/L) in the children with glucocorticoid treatment were significantly higher than in those without glucocorticoid treatment (560 nmol/L, 41 to 8,069 nmol/L; *P *< 0.01); blood glucose levels did not differ.

##### Influence of etomidate

Thirty-five of the children were intubated and had received a single dose of etomidate. As we have previously shown that use of etomidate negatively influenced blood glucose levels, we assessed the influence of etomidate. The children who had received etomidate showed significantly lower glucose and cortisol levels (6.2 mmol/L, 4.7 to 8.5 mmol/L and 713 nmol/L, 555 to 958 nmol/L, respectively) on admission in comparison with the other children (7.7 mmol/L, 5.6 to 10.0 mmol/L and 1,133 nmol/L, 953 to 1,342 nmol/L, respectively; *P *< 0.01). At 24 hours after admission, blood glucose levels in etomidate-treated children were significantly higher than in the others (7.2 mmol/L versus 6.6 mmol/L; *P *= 0.03), presumably because of a rebound effect. Multiple regression analysis showed that the insulin and age effect on blood glucose levels as described in section "Correlations" was not influenced by etomidate administration.

### Correlations

Blood glucose levels correlated positively with plasma insulin levels (Figure 1; *r *= 0.67, *P *< 0.001), C-peptide levels (*r *= 0.46, *P *< 0.01), cortisol levels (*r *= 0.27, *P *< 0.05), and age (*r *= 0.43, *P *< 0.001). Multiple regression analysis revealed that both age and plasma insulin levels on admission were factors positively related to blood glucose level (*P *= 0.035 and *P *< 0.001, respectively). These two variables together explained 41% of the variance in blood glucose level on admission. The other variables (glucose intake, cortisol level, [nor]-adrenaline therapy, and steroid use) were not significantly related to blood glucose level on admission. The two outcome parameters, HOMA-%S and insulin-to-glucose ratio, were significantly correlated (*r *= 0.87, *P *< 0.001). C-peptide levels were strongly correlated with insulin levels (*r *= 0.82, *P *< 0.001).

## Discussion

Thirty-three percent of all children in the present study were hyperglycemic on admission, and one child was hypoglycemic. Blood glucose levels in shock and sepsis survivors were higher than in shock non-survivors. Hyperglycemic children had significantly higher insulin and C-peptide levels in comparison with normoglycemic children. HOMA showed a predominance of insulin resistance in hyperglycemic children, although β-cell insufficiency or a combination of insulin resistance and β-cell insufficiency was also seen. Multiple regression analysis revealed that both age and plasma insulin levels on admission were significantly related to blood glucose level.

Hyperglycemia is a common finding in critically ill children, and our results are in line with those of previous studies [8,11,14]. Whereas others have reported an association between hyperglycemia and mortality [8-14], we showed, in the present study, that shock non-survivors had the lowest blood glucose levels. This study concerns children with meningococcal sepsis and septic shock, whereas the other studies included children with mixed diagnoses. Only Branco and colleagues [12] studied children with septic shock (various causes) and showed that a peak glucose level of greater than 9.8 mmol/L was independently associated with an increased risk of death (relative risk of 2.59).

In our study, insulin levels on admission were the lowest in children who did not survive and were closely related to the low blood glucose levels. The association between a lower blood glucose level on admission and mortality in the present study might be explained by the specific features of meningococcal disease, like the high risk for relative adrenal insufficiency [5]. This could also explain the positive correlation between blood glucose levels and age, as the youngest children showed the highest mortality rate in combination with the lowest blood glucose levels on admission. Previously, we showed that the concomitant use of therapeutic drugs such as etomidate, which was used in almost half of the studied children, influenced blood glucose levels as well [5]. In accordance with previous findings, children intubated with etomidate showed lower glucose and cortisol levels on admission in comparison with those without etomidate. Hyperglycemia was associated with elevated insulin levels in half of the children. HOMA showed that insulin resistance as well as β-cell dysfunction resulting in a hypoinsulinemic response resulted in hyperglycemia. Insulin resistance, caused by high levels of counter-regulatory hormones and cytokines, oxidative stress, and therapeutic interventions (such as glucocorticoid and catecholamine administration), is the main pathophysiological mechanism of hyperglycemia in critically ill patients [32].

Concerning therapeutic interventions, glucocorticoid and catecholamine use in insulin-resistant hyperglycemic children was more frequent than in those without insulin resistance. However, the numbers were too small to detect significant differences. Cortisol level on admission was positively correlated with plasma glucose level in children without previous glucocorticoid treatment, indicating that endogenous cortisol release is a causative factor for hyperglycemia. Sepsis guidelines recommend glucocorticoids for the treatment of vasopressor-dependent septic shock [15]. Glucocorticoids stimulate hepatic glucose production, mainly by mobilizing substrate for hepatic gluconeogenesis and activation of key hepatic gluconeogenic enzymes. Furthermore, glucocorticoid excess reduces glucose uptake and utilization by peripheral tissues, owing in part to direct inhibition of glucose transport into the cells [33]. Hyperglycemic episodes were more common in adult septic shock patients who received hydrocortisone in bolus therapy as compared with those who received a continuous infusion with an equivalent dose [34]. This important side effect of glucocorticoid treatment has not yet been addressed in studies in critically ill children.

Another important causative factor of hyperglycemia might be the amount of glucose intake. In the present study, children were considered to be fasting on admission, because they received only a continuous glucose infusion without enteral intake. Glucose intake did not differ between normoglycemic and hyperglycemic children. In critically ill adults, an association between hyperglycemia and a high glucose infusion rate (greater than 5 mg/kg per minute) was shown [35]. On the other hand, low-caloric parenteral nutrition in adult surgical trauma patients resulted in fewer hyperglycemic events and lower insulin requirements [36]. Maximum glucose oxidation rates in severely burned children approximate 5 mg/kg per minute [37]. Exogenous glucose in excess of this amount enters non-oxidative pathways and is unlikely to improve energy balance and lipogenesis and may result in hyperglycemia [38,39].

Two studies have suggested that a hypoinsulinemic response in critically ill children might result in hyperglycemia [18,40]. First, van Waardenburg and colleagues [18] studied 16 children with meningococcal disease on the third day of admission (10 shock survivors and 6 sepsis survivors). Whereas most children were normoglycemic, shock survivors had lower insulin levels (50 pmol/L) and insulin-to-glucose ratios (8 pmol insulin per mmol glucose) in comparison with sepsis survivors (130 pmol/L and 24 pmol insulin per mmol glucose, respectively), suggesting normal or enhanced insulin sensitivity in shock survivors. Second, Preissig and Rigby [40] showed relatively low C-peptide levels (1.5 nmol/L, 4.4 ng/mL) within 48 hours after admission in hyperglycemic critically ill children with respiratory and cardiovascular failure. Accordingly, the present study also showed relatively low C-peptide levels for shock survivors and sepsis survivors during admission (1.0 to 1.7 nmol/L, 3.0 to 5.1 ng/mL). HOMA-%B based on paired C-peptide, insulin, and glucose levels showed β-cell dysfunction of the pancreas in 38% of hyperglycemic children who were either shock or sepsis survivors. The cause of pancreatic dysfunction could have many factors, including elevations in pro-inflammatory cytokines, catecholamines, and glucocorticoids. It was hypothesized that β-cells become dysfunctional if physiological changes occur acutely. When the same changes occur more gradually, this might allow β-cells to adapt and function at supraphysiological levels over time, resulting in insulin resistance. Also, β-cell exhaustion is a known phenomenon characterized by an ability to increase secretion up to a certain level and thereafter fail in response to further demand.

Finally, proinflammatory cytokines are important mediators of the hyperglycemic stress response. We did not find correlations between cytokines and insulin levels or HOMA-%S in hyperglycemic children, presumably because of the relatively small sample size.

Forty-eight hours after admission, the percentage of children with hyperglycemia had decreased from 33% to 8% without insulin therapy. In contrast, in critically ill adult patients, hyperglycemia may persist for days to weeks with or without insulin therapy [41]. This difference might be due to the rapid resolution of the acute stress response that is seen in severely ill children with meningococcal disease [5]. The present data also show that the elevated cortisol and cytokine levels on admission decrease to normal values within 24 hours.

There are several limitations to this study. The hyperinsulinemic euglycemic clamp technique is the 'gold standard' for quantifying insulin sensitivity *in vivo *because it directly measures the effects of insulin to promote glucose utilization under steady-state conditions. It is not easily implemented, however, in large studies with critically ill children. In the present study, therefore, insulin sensitivity was indirectly assessed by investigating the insulin response to glucose and by HOMA. Diabetes studies and epidemiological studies on glucose tolerance have frequently used HOMA, and recent reports have shown its value for assessment of insulin sensitivity in the critically ill [22,23]. Nevertheless, as we are the first to use HOMA analysis to describe insulin resistance and β-cell dysfunction in critically ill children, there are no control data for HOMA for sick children and we have to be careful in our conclusions. Under basal conditions, the product of β-cell responsivity and insulin sensitivity is assumed to be a constant, and different values of tolerance are represented by different hyperbolas [42]. We have shown that, in critically ill children with impaired glucose tolerance, β-cells can be dysfunctional, resulting in an inadequate compensatory increase in insulin release to the decreased insulin sensitivity.

## Conclusions

Hyperglycemia with a blood glucose level of greater than 8.3 mmol/L on admission is frequently seen in children with meningococcal sepsis and septic shock; hypoglycemia is also seen but less frequently. Blood glucose levels in most children spontaneously normalize within 48 hours, at normal glucose intake and without insulin treatment. Both insulin resistance as well as β-cell dysfunction may contribute to the occurrence of hyperglycemia in critically ill children with meningococcal sepsis and septic shock.

## Key messages

• Hyperglycemia with a blood glucose level of greater than 8.3 mmol/L (greater than 150 mg/dL) on admission is seen in 33% of critically ill children with meningococcal disease.

• Pathophysiologically, both a hyperinsulinemic and a hypoinsulinemic response play a role in the occurrence of hyperglycemia in critically ill children with meningococcal disease.

• Critically ill children with hyperglycemia can be classified, on the basis of blood glucose level and HOMA-%S and HOMA-%B, into those with overt insulin resistance and those with decreased β-cell function.

• Children with meningococcal septic shock who do not survive have the lowest levels of blood glucose and insulin levels compared with those who survive.

• In children with meningococcal disease, normalization of blood glucose levels occurs within 48 hours, typically with normal glucose intake and without insulin treatment.

## Abbreviations

APC: activated protein C concentrate; CRP: C-reactive protein; FFA: free fatty acid; HOMA: homeostasis model assessment; HOMA-%B: homeostasis model assessment of β-cell function; HOMA-%S: homeostasis model assessment of insulin sensitivity; IL-6: interleukin-6; PICU: pediatric intensive care unit; PO_2_: partial pressure of oxygen; PRISM: pediatric risk of mortality.

## Competing interests

The authors declare that they have no competing interests.

## Authors' contributions

JV performed literature searches and statistical analysis and wrote this paper under the direct supervision of KJ. MdB participated in the coordination of the study and carried out the data collection. AH-K participated in the design of the study and helped to edit and revise the paper critically. JH participated in the design and coordination of the study and helped to draft the manuscript. KJ conceived of the study, participated in its design and coordination, and helped to draft the manuscript. All authors read and approved the final manuscript.
